# Combining Electrochemical Sensors with Miniaturized Sample Preparation for Rapid Detection in Clinical Samples

**DOI:** 10.3390/s150100547

**Published:** 2014-12-30

**Authors:** Natinan Bunyakul, Antje J. Baeumner

**Affiliations:** 1 Department of Clinical Chemistry, Faculty of Medical Technology, Mahidol University, Nakhon Pathom 73170, Thailand; E-Mail: natinan.bun@mahidol.ac.th; 2 Institute of Analytical Chemistry, Chemo- and Biosensors, University of Regensburg, Regensburg 93053, Germany; 3 Department of Biological and Environmental Engineering, Cornell University, Ithaca, NY 14853, USA

**Keywords:** electrochemical sensor, microfluidic-based sample preparation, clinical sample analysis

## Abstract

Clinical analyses benefit world-wide from rapid and reliable diagnostics tests. New tests are sought with greatest demand not only for new analytes, but also to reduce costs, complexity and lengthy analysis times of current techniques. Among the myriad of possibilities available today to develop new test systems, amperometric biosensors are prominent players—best represented by the ubiquitous amperometric-based glucose sensors. Electrochemical approaches in general require little and often enough only simple hardware components, are rugged and yet provide low limits of detection. They thus offer many of the desirable attributes for point-of-care/point-of-need tests. This review focuses on investigating the important integration of sample preparation with (primarily electrochemical) biosensors. Sample clean up requirements, miniaturized sample preparation strategies, and their potential integration with sensors will be discussed, focusing on clinical sample analyses.

## 1. Introduction to Biosensor

Since the first biosensors were proposed and demonstrated by Clark and Lyons in 1962 [1], the idea behind biosensors has been explored in a wealth of variations and has been defined with specific criteria by international union of pure and applied chemistry (IUPAC) in 1999 [2]. The exquisite specificity and sensitivity of biological recognition elements including antibodies [3], oligonucleotides [4], enzymes [5], and cell receptors [6] transduced through physical and chemical strategies that are not limited to electrochemical, optical or mass-based means has led to amazing analytical systems. The electrochemical glucose biosensor based on Clark's original concept is the best known, likely best studied, and surely commercially most successful biosensor to date [7,8]. As much as new sensing systems are being developed today, effort is also put toward the important aspect of integration of the detection system with an efficient and appropriate sample preparation strategy to deal with actual real-world samples. Here, great expectations are put toward miniaturized “total analysis systems” (microTAS) that hold the promise of integrating sample preparation and biosensing in one small chip, creating a portable device.

Electrochemical biosensors lend themselves well to clinical analysis as demonstrated exemplary by successful glucose sensors, the iStat, and other chemical sensors for blood gas and ion analysis [9,10]. The low-tech hardware requirements and high sensitivity are two major advantages that lead to the abundance of electrochemical biosensors. Transduction principles seen in clinical analysis include primarily amperometry, cyclic voltammetry, and differential pulse voltammetry. In addition to these electrochemical sensors, clearly no shortage of detection principles and assay formats exists ranging from optical, to mass-based, and piezoelectric formats [11], each providing unique aspects that are advantageous for specific settings, relating to limits of detection, ease-of-use, costs, assay time and alike.

The range of analytes relevant in clinical diagnostics that have been addressed by biosensors and bioanalytical systems (not limited to electrochemical transduction) is staggering [12], including cancer, genome analysis, autoimmune diseases, infectious diseases, and cardiac biomarkers. In the case of infectious disease applications, monitoring and diagnostics of pathogenic microorganisms has been described for a long list of analytes (Table 1) also including those analytes that are relevant to the food industry, water, and environmental applications [13]. Maybe not surprisingly, the typical common challenge of biosensors that are designed for application to real-world samples is the matrix of the specimens, which may likely interfere with the results or negatively affect the detection principle of the assay. In the case of clinical specimens, such as blood (whole blood, serum, or plasma), urine, saliva, stool, sputum, and tissue, this challenge of sample preparation for diagnostics has been described by J. Liao and his group recently [14]. How miniaturized biosensors solve these challenges will be addressed further along in this article.

## 2. Pairing (Electrochemical) Biosensors with Sample Preparation for Analyte Detection in Clinical Samples

Significant effort has to be invested in the design of a biosensor so that it can be applied to actual real-world samples. It is well known and often described how matrix effects, non-specific binding and interferences will negatively affect a biosensor signal to the point that no qualitative or quantitative analysis is possible. Sensor surfaces are therefore typically protected via membranes, films or simple blocking layers of adsorbed molecules in order to prevent any of these interferences. Examples are the polyethylene glycol modified membrane of glucose sensors that prevent components such as ascorbic acid and uric acid to reach the electrode surface and hence render the electrochemical transduction specific [20,21]. Also, in heterogeneous immunoassays, surfaces are blocked with polymers or proteins, such as polyvinylpyrrolidone [22,23], gelatin [22,24] casein [25,26], or bovine serum albumin [27,28], respectively. Hydrogels or similar films are often applied to not only immobilize the biorecognition element but also function as diffusion barrier for interferences from the matrix [29,30].

However, coatings and blocking strategies cannot circumvent all negative sample matrix effects, including fouling of surfaces, interference with biorecognition reactions, clogging of fluid channels, *etc.*, and sample preparation is hence of imminent importance. Different criteria apply for different transduction principle in order to avoid matrix-effects. For example, turbidity is a common problem for optical sensors, auto-fluorescence for any fluorescence-based system, non-specific adherence of any particle is a challenge for mass-based systems, and the avoidance of electrochemically active compounds is mandatory for electrochemical sensors. In Table 2 specific criteria for sample preparation processes are listed as they relate to applications of clinical sample analyses with electrochemical sensors and those when used in microfluidic systems.

**Figure 1.**
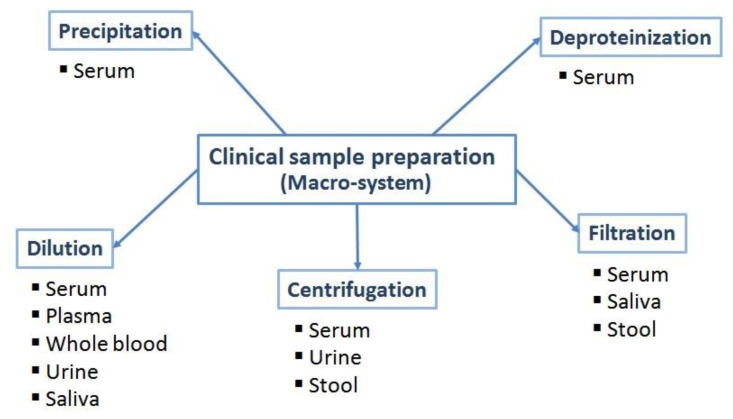
Summary of the most often applied macro-system sample preparation procedures for clinical samples.

The most often applied sample preparation steps are summarized in Figure 1. Whenever possible, the sample is being diluted in order to shift the effect of interferences below a tolerable threshold, *i.e.*, when blocking and protecting functionalities of the biosensor design can be effective against undesired matrix components. This has been demonstrated, for example with glucose analyzers, such as those developed by Yellow Springs Instrument Company (Yellow Springs, OH, USA). Glucose oxidase is immobilized between two membrane layers. The outer polycarbonate membrane retains the enzyme, allows glucose to pass, but prevents larger molecules from entering, thus reducing interferences. The inner membrane is gas selective and necessary for the selectivity of the sensor [43]. Another example is the multilayered membranes developed by Matsumoto *et al.*, which are able to measure glucose concentrations in a high enough range so that no sample dilution is required. Furthermore, the sensor provides a rapid response, a wide measuring range, and practical immunity to interference species (ascorbic acid, uric acid, and p-acetaminophen) [44,45]. However dilution or thick protective layers are obviously only applicable, if the analyte is present at high enough concentrations. Instead, other, frequently used simple sample preparation procedures include centrifugation, filtration, precipitation and deproteinization.

Blood as clinical sample has the advantage that it is the most rich with respect to variety of relevant analytes, yet also has the disadvantage to be the most rich with respect to matrix complexity and viscosity [14]. It can be divided into three types of specimen for each of which many amperometric biosensors have been presented, *i.e.*, whole blood [46,47], serum [48–58], and plasma [59–61]. For whole blood and plasma, dilution is the most frequently used sample preparation step and was, for example used for the analysis of Zn^2+^ [46], neuropathy target esterase [47], glucose [20], pyrazinamide [59], prostate specific antigen [60], and nitrite/nitrate [61].

Similarly, also for serum samples, dilution is the most often utilized technique and is combined with additional processing steps, such as centrifugation for dopamine [48], uric acid [48], glucose [49], and immunoglobulin A [62] analysis; precipitation for dopamine [53] and biphenyl [58] analysis; deproteinization with acid and filtration for glucose [55] analysis. It is important to keep in mind, though, that in some instances, especially in single-use devices, biosensors are described that can deal with the complex blood matrix without sample pretreatment step such as shown for glucose where Nafion membranes are known to cut down the most prevalent interferences such as ascorbic and uric acid [63] and nucleic acids (miRNAs) [64].

In the case of urine samples, the wide range of pH values found in samples can be challenging [14]. In addition to pH adjustment, centrifugation and dilution are two of the most often used sample preparation techniques as described for analytes, such as pirazinamide [58], anti-malarial drug (Artesunate) [65], testosterone [66], homocysteine [67], nuclear matrix protein 22 [68], dopamine [69], and uric acid [70–72].

Similar to blood, saliva samples suffer from an immense component complexity and variation of compositions. Here, filtration and dilution methods are for example utilized for lactate [73] and nitrite/nitrate [62] analysis, respectively.

Challenges associated with stool samples are most prominently similar to those of other solid materials such as soil, and solid food samples, but also the presence of high concentrations of bile. Centrifugation or filtration is typically a must in order to remove particulates, especially when considering microfluidic sensor developments [74].

## 3. Recent Strategies of Miniaturized Sample Preparation and Their Comparison to Bench-Top Standards

**Figure 2.**
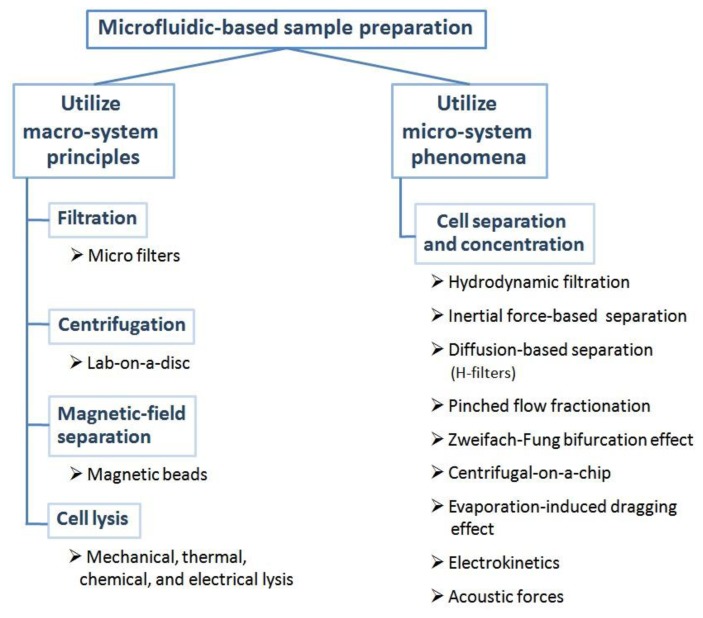
Summary of microfluidic-based sample preparation techniques that are classified into two groups: (1) those obtained by scaling down a macro-system and (2) utilization of micro-system phenomena.

When miniaturizing biosensors for clinical analysis, requirements for and necessity of analyte isolation from the sample matrix remain of utmost importance, in fact, additional challenges are added (Table 2). Microfluidic-based sample preparation can be classified into two groups (Figure 2). Most simply put, microtechniques are developed that copy one-to-one those techniques found in the macro-system, alternatively micro-phenomena are exploited to produce the same sample preparation result. The comparison of microtechniques with corresponding bench-top strategies (Table 3) can be done either by directly comparing performance characteristics or by comparing final limits of detection reported for the respective target or model analytes. In some cases, this comparison is straight forward based on published data, in other cases this is more challenging due to limited data available. This section provides a few case studies for these important comparative evaluations.

With respect to microfluidic techniques that utilizing macro-principles, filtration is an excellent example, such as the filtration of red blood cell agglutination complexes via paper-based microfluidics in order to detect the target analyte present in the plasma [75]. Microfilters [86–88] have also been developed as the straightforward method for cell separation in micro-system. Alternatively, centrifugation has been realized using lab-on-a-disc for the separation of target cells [78]. Similarly, magnetic field separation is realized in micro-systems by bead-based analyte capture integrated with microfluidic systems [89,90]. Cell lysis techniques used in the macro-system have also been realized in micro devices, such as mechanical [40,91], thermal [92–94], chemical [95], and electrical lysis [96]. All of these techniques can reduce the volume of sample/reagent, which is the main advantage of the scaling down devices while keeping the scientific principle of the sample preparation step the same.

Comparing their efficiency to standard bench-top methods has been described by some researchers. An excellent example is the use of microfilter membranes for cell separation or concentration in microdevices. Yang *et al.* [75] developed a paper-based microfilter membrane for the separation of plasma from whole blood with the purpose of plasma glucose determination using a glucose oxidase-based colorimetric assay. The researchers compared this sample preparation technique with the conventional centrifugation method (800 rcf, 15 min) and found a good correlation of the results for both techniques. Similarly, parylene microfilter membranes, which were developed by Lin *et al.* [76], were applied to the identification of circulating tumor cells (CTCs) in whole blood. This system was shown to achieve more than 90% recovery and in fact showed better CTC identification when compared with CellSearch, a bench-top immunomagnetic separation technique.

Also for magnetic bead and centrifugal force principles, the scaling down resulted in comparable results. For example, a magnetic bead-based proximity ligation assay was developed in which magnetic field-enhanced separation of the target analyte from human plasma was performed [77]. The detection range of this micro-system was found to be at 5–100 pg/mL. This compared well with respect to the limit of detection of a bench-top ELISA (2.2–50,000 pg/mL) for TNF-quantification, but fell short with respect to the dynamic range achievable. Lee *et al.* [78] developed a disc-based assay for anti-HBs and HBsAg from whole blood utilizing centrifugal forces for fluid movements. Their “Lab-on-a-disc” technique demonstrated comparable limits of detection to a bench-top ELISA for both analytes.

As final example, cell lysis [40] using a miniaturized magnetically actuated bead-beating system was compared to the standard in-tube bead beating lysis method. In both cases, centrifugation and RT-PCR followed the initial lysis step for the detection of respiratory pathogens in nasopharyngeal aspirates. No difference in lysis efficiency was found between the micro- and macro systems.

**Figure 3.**
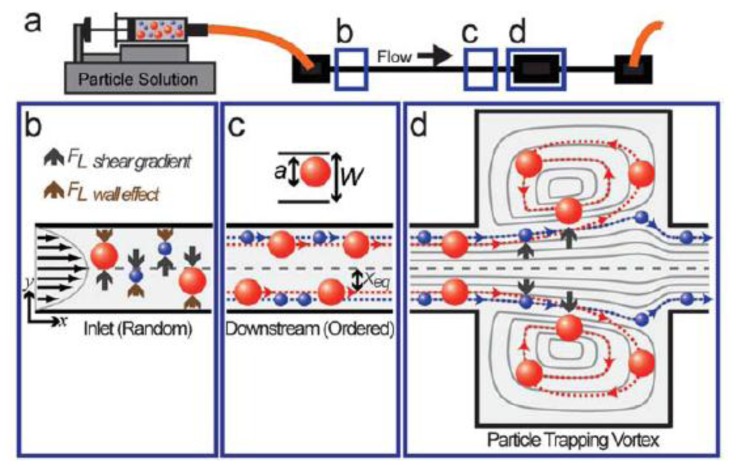
Particle entry mechanism in laminar microvortices. (**a**) For a polydisperse particle solution injected into a device with a straight high aspect ratio channel leading into an expansion-contraction chamber we expect size-dependent entry into the laminar vortices created; (**b**, **c**) Particles are subjected to a shear gradient lift force, which directs particles toward the channel wall, and a wall effect lift force, directed toward the channel center, which leads to entrainment of particles at dynamic equilibrium positions, X_eq_; (**d**) As focused particles enter the vortex chamber, the lift forces are decoupled due to the absence of a nearby wall, resulting in a dominate shear gradient lift force. Larger particles (red) experience larger lift forces and are able to migrate across fluid streamlines into the vortices while smaller particles (blue) follow fluid streamlines and flow out of the system [103] with permission of The Royal Society of Chemistry.

The second strategy to realize sample preparation in a miniaturized system takes advantage of phenomena unique to microfluidic systems or utilizes those that are very easy to realize in the micro-world in comparison to the macro-system. For example, cell separation and concentration can be accomplished using hydrodynamic phenomena, such as the Zweifach-Fung bifurcation effect [97,98], inertial force-based cell separation [99–102], centrifugal-on-a-chip (Figure 3) [103], evaporation-induced dragging effect [104], hydrodynamic filtration [105,106], pinched flow fractionation [107,108], and diffusion-based cell separation by using H-filters [109]. Cell separation has also been demonstrated using active separation techniques such as electrokinetic strategies [110–113] and acoustic forces [84,114].

From a microfluidic device development point of view, the use of “microfluidic phenomena” comparability of results is very important, as completely new parameters are applied in bench-top and microsystems. Following are a few interesting studies reported. For example, for the separation of cancer cells from whole blood, an inertial force-based method was developed [79] and compared with flow cytometry. The microdevice showed superb cancer cell recovery rates in whole blood of 99.1%, blood cell rejection ratio of 88.9%, and a throughput of 1.1 × 10^8^ cells/min which is comparable to the commercial flow cytometry systems' achieved throughput (∼2.4 million cells/min) [115]. The same inertial force-based technique was also applied for neural cell separation from cell culture medium [80]. Here, a throughput of ∼1 million cells/min was found to be comparable to the commercial macroscale flow cytometer with an 80% efficiency and high relative viability (>90%).

When comparing dielectrophoresis with macro-system centrifugation for blood plasma separation [81], plasma yield of 15.6% ± 2.5% and purity efficiency of 94.2% ± 3.6% were found for dielectrophoresis and plasma yield of 95% and purity efficiency of 99% were found for the centrifugation technique. Blood plasma separation by other microfluidic-based methods was also studied. Plasma yield of 40% and purity efficiency of 53% were found for the development of blood plasma separation by using the Zweifach-Fung effect [82] and 80% of erythrocyte separation efficiency was found for the development of a Pinched-flow fractionation [83] microdevice. In other cases, lipid particle separation from blood was investigated which are relevant for intra-operative blood wash applications [84]. Here, Petersson *et al.* utilized an acoustic force-based technique and removed more than 80% of the lipid particles from the blood while collecting ∼70% of the erythrocytes (recovery). The researchers discussed the quality of the separation to be excellent and additionally avoid standard problems of macroscale wash steps based on centrifugation including hemolysis, discontinuity, and a demand for large volumes (∼500 mL) of blood.

The Yager research group [85] developed an H-filter diffusion-based technique for the separation of small molecular analytes (Phenytoin, 252 Da) from saliva samples. The H-filters were comparable to centrifugal techniques [85,116], which were used to extract the analyte from the remaining large molecular weight species in the filtered saliva sample. Specifically, the H-filter processed saliva sample retained 23% of the analyte with 97% and 92% reduction in glycoproteins and proteins, respectively. Furthermore, subsequent detection processes were improved as the H-filter processed sample caused significantly less fouling of biosensor surfaces.

Gillers *et al.* [117] developed microfluidic-based DNA extraction from crude stool samples prior to PCR amplification. While no direct comparison to the bench-top DNA extraction method was provided, the authors could demonstrate that their on-chip method resulted in extract purity suitable for subsequent PCR.

## 4. Conclusions

Bioanalytical sensors and miniaturized sample preparation strategies have been described and successfully applied to a variety of clinical samples. We conclude that the combination of several of the miniaturized sample preparation assays are ideally suited for the integration with electrochemical detection strategies. For example, the above-described acoustic force-based technique used for the separation of lipid particles [84] can easily be combined with a simple miniaturized amperometric detection strategy [74]. Here, electrochemical sensors such as those using nanomaterials integrated with the screen-printed electrodes (SPE) surface for cardiac biomarkers [118,119] will benefit from such a sample preparation step as electrode fouling through lipid particles will be avoided. Similarly, the dielectrophoretic generation of plasma from blood samples [81] would mean that plasma tests performed for human health diagnosis and treatment can be performed by simply applying the finger tip's whole blood sample onto the microfluidic device and waiting for the results (sample-to-answer concept) [120]. In addition, saliva samples can be prepared and analyzed within microdevices for the detection of antibodies to HIV, therapeutic drugs and steroids [121] if an H-filter diffusion-based separation technique is directly integrated on chip.

Assay systems like these can overcome the greatest shortcoming of today's bioanalytical detection systems and be developed into commercially viable diagnostic tests. They will be effective, simple and rugged self-contained assays for point-of-care and point-of-need testing that on the one hand integrate innovative and novel concepts and on the other hand rely on well-established concepts that can be trusted for clinical diagnostics.

## Figures and Tables

**Table 1.** Summary of pathogenic organisms relevant to clinical diagnostics for which biosensors have been developed.

**Virus**	**Bacteria**	**Fungi**
VariolaV [15,16]	*Rickettsia prowazecki* [15,16]	*Coccidioidesimmitis* [15,16]
ChikungunyaV [15,16]	*Rickettsia rickettsi* [15,16,19]	*Histoplasmacapsulatum* [15,16]
Eastern encephalitis V [15,16]	*Rickettsia tsutsugamushi* [15,16]	*Nocardiaasteroides* [15,16]
Venezuelan encephalitis V [15,16]	*Bacillus anthracis* [15,16,19]	
Western encephalitis V [15,16]	*Francisella (Pasteurella)tularensis* [15,16,19]	
Dengue V [15,16]	*Pasteurellapestis* [15,16]	
Yellow fever V [15,16]	*Brucellamelitensis, B. suis* [15,16,19]	
Japanese encephalitis V [15,16]	*Coxiellaburnetti* [15,16,19]	
Russian spring-summer encephalitis V [15,16]	*Salmonella typhi* [15–17,19]	
Argentine hemorrhagic fever V [15,16]	*Salmonella paratyphi* [15,16,19]	
Lassa fever V [15,16]	*Salmonella enteric* [17]	
Lymphocyte choriomeningitis V [15,16]	*Shigelladysenteriae* [19].	
Bolivian hemorrhagic fever V [15,16]	*Vibrio cholerae* [15–17,19]	
Crimean-Congo hemorrhagic fever V [15,16]	*Corynebacterium diphtheria* [15,16,19]	
Haantan (Korean hemorrhagic fever) V [15,16]	*Actinobacillus mallei* [15,16]	
Rift Valley fever V [15,16]	*Pseudomonas aeruginosa* [17]	
Marburg V [15,16]	*Pseudomonas pseudomallei* [15,16]	
Ebola V [15,16]	*Mycobacterium tuberculosis* [15,16,19]	
Hepatitis (A, E) V [15–17]	*Burkholderiapseudomallei* [17]	
Norwalk V [18]	*Campylobacter jejuni* [17,19]	
	*Clostridium botulinum* [19]	
	*Escherichia coli*-pathogenic [17,19]	
	*E. coli* O157: H7 [18]	
	*Legionella* spp. [17]	
	*Yersinia enterocolitica* [17]	
	*Yersinia pestis* [19]	
	*Treponemapallidum* [19]	
	*Streptococcus pneumonia* [19]	
	*Staphylococcus aureus* [19]	
	*Listeria monocytogenes* [18]	

**Table 2.** Important criteria for sample preparation processes considerations for the development of electrochemical (micro) sensors.

**Criteria Specific to Electrochemical Sensors**	**Examples**	**Additional Important Criteria and Those Specific to Microfluidic Electrochemical Sensors**	**Examples**
Removal of electrochemically active compounds	*In serum/plasma* [31]: - Uric acid - Ascorbic acid - Dopamine - L-cysteine - Acetaminophen - Salicylic acid *In urine* [32]: - Urea - Tartaric acid - Citric acid - Glucose - Leucine - Proline - Tyrosine *In saliva* [33]: - Uric acid - Ascorbic acid	Removal of particulate to avoid clogging of microchannels and microvalves [34]	Blood cells may form aggregates clogging the microchannels during separation of plasma from blood [35]

Adjustment of ionic strength and temperature [36,37]	- Variable ionic strength influence potentiometric, conductimetric and also voltammetric measurements. In addition, ionic strength and nature affects biological reactions [36] - Temperature affects the slope of the electrode response according to the Nernst equation [37]	Reducing non specific absorption of hydrophobic material such as PDMS [38]	Adsorption of fluorescence markers can cause a drift in the background fluorescence intensity [38]

Removal of surface fouling compounds [39]	Fouling cause by plasma proteins, lipids, and other biochemical components of the biological fluids [39]	Removal of compounds interfering with the biorecognition or signal amplification mechanisms [40]	PCR inhibitors in blood sample such as heme, hemoglobin, lactoferrin and immunoglobulin G [40]

Adjustment of pH [41]	A pH buffer can be used to reduce hydroxyl ion (OH^−^) effects that interfere ISE electrodes [41]	Adjustment of pH [42]	Surface charge (Zeta potential) of the microchannels' walls is generally a function of the pH thus, the electroosmotic pumping process can be enhanced or degraded by changes in pH [42]

**Table 3.** Comparison between micro techniques to corresponding bench-top methods for sample preparation based on published data.

**On-Chip Sample Preparation Techniques**	**Bench-Top Methods**	**Comparison Result of On-Chip To the Bench-Top Method**	**References**
Microfilter membrane (Paper-based)	Centrifugation	Comparable	[75]
Microfilter membrane (Parylene)	Immunomagnetic separation	Better	[76]
Magnetic bead-based separation	ELISA	Comparable	[77]
Lab-on-a-disc	ELISA	Comparable	[78]
Miniaturized bead-beating	In-tube bead-beating	Comparable	[40]
Inertial force-based	Flow cytometry	Comparable	[79,80]
Dielectrophoresis	Centrifugation	Comparable (for purity)	[81]
Zweifach-Fung bifurcation	Centrifugation	Worse	[82]
Pinched- flow fractionation	Centrifugation	Worse	[83]
Acoustic force-based	Centrifugation	Better	[84]
Diffusion-based (H-filter)	Centrifugation	Comparable	[85]

## Abbreviations

CTCs: circulating tumor cells
ELISA: enzyme-linked immunosorbent assay
IUPAC: international union of pure and applied chemistry
microTAS: micro total analysis systems
RT-PCR: reverse transcription polymerase chain reaction
SPE: screen-printed electrodes
TNF: tumor necrosis factors

## References

[1] Clark L.C., Lyons C. (1962). Electrode systems for continuous monitoring in cardiovascular surgery. Ann. N. Y. Acad. Sci..

[2] Thévenot D.R., Toth K., Durst R.A., Wilson G.S. (2001). Electrochemical biosensors: Recommended definitions and classification. Biosens. Bioelectron..

[3] Luppa P.B., Sokoll L.J., Chan D.W. (2001). Immunosensors-principles and applications to clinical chemistry. Clin. Chim. Acta.

[4] Tosar J.P., Brañas G., Laíz J. (2010). Electrochemical DNA hybridization sensors applied to real and complex biological samples. Biosens. Bioelectron..

[5] Valdés-Ramírez G., Cortina M., Ramírez-Silva M.T., Marty J.-L. (2008). Acetylcholinesterase-based biosensors for quantification of carbofuran, carbaryl, methylparaoxon, and dichlorvos in 5% acetonitrile. Anal. Bioanal. Chem..

[6] Singh A.K., Harrison S.H., Schoeniger J.S. (2000). Gangliosides as receptors for biological toxins: Development of sensitive fluoroimmunoassays using ganglioside-bearing liposomes. Anal. Chem..

[7] Wang J. (2008). Electrochemical glucose biosensors. Chem. Rev..

[8] Palmisano F., Zambonin P.G., Centonze D., Quinto M. (2002). A disposable, reagentless, third-generation glucose biosensor based on overoxidizedpoly(pyrrole)/tetrathiafulvalene-Tetracyanoquinodimethane composite. Anal. Chem..

[9] D'Orazio P. (2003). Biosensors in clinical chemistry. Clin. Chim. Acta.

[10] Yoo E.-H., Lee S.-Y. (2010). Glucose biosensors: An overview of use in clinical practice. Sensors.

[11] Justino C.I.L., Rocha-Santos T.A., Duarte A.C., Rocha-Santos T.A. (2010). Review of analytical figures of merit of sensors and biosensors in clinical applications. TrAC Trends Anal. Chem..

[12] D'Orazio P. (2011). Biosensors in clinical chemistry—2011 update. Clin. Chim. Acta.

[13] Lazcka O., Campo F.J.D., Muñoz F.X. (2007). Pathogen detection: A perspective of traditional methods and biosensors. Biosens. Bioelectron..

[14] Sin M.L., Mach K.E., Wong P.K., Liao J.C. (2014). Advances and challenges in biosensor-based diagnosis of infectious diseases. Expert Rev. Mol. Diagn..

[15] Gander T.J. (1992). Chemical and biological warfare agents. Jane's NBC Protection Equipment.

[16] Gessler E. (1986). Biological and Toxin Weapons Today.

[17] World Health Organization (2008). Guidelines for Drinking-Water Quality.

[18] Sharma H., Mutharasan R. (2013). Review of biosensors for foodborne pathogens and toxins. Sens. Actuators B Chem..

[19] Ivnitski D., Abdel-Hamid I., Atanasov P., Wilkins E. (1999). Biosensors for detection of pathogenic bacteria. Biosens. Bioelectron..

[20] Sun C., Miao J., Yan J., Yang K., Mao C., Ju J., Shen J. (2013). Applications of antibiofouling PEG-coating in electrochemical biosensors for determination of glucose in whole blood. Electrochim. Acta.

[21] Sung W.J., Na K., Bae Y.H. (2004). Biocompatibility and interference eliminating property of pullulan acetate/polyethylene glycol/heparin membrane for the outer layer of an amperometric glucose sensor. Sens. Actuators B Chem..

[22] Martorell D., Siebert S.T.A., Durst R.A. (1999). Liposome dehydration on nitrocellulose and its application in a biotin immunoassay. Anal. Biochem..

[23] Studentsov Y.Y., Schiffman M., Strickler H.D., Ho G.Y.F., Susana Pang Y.-Y., Schiller J., Herrero R., Burk R.D. (2002). Enhanced enzyme-linked immunosorbent assay for detection of antibodies to virus-like particles of human papillomavirus. J. Clin. Microbiol..

[24] Székács A., Le H.T.M., Szurdoki F., Hammock B.D. (2003). Optimization and validation of an enzyme immunoassay for the insect growth regulator fenoxycarb. Anal. Chim. Acta.

[25] Wu J.T., Zhang P., Liu G.H., Wilson L. (1998). Development of an immunoassay specific for the PSA-ACT complex without the problem of high background. J. Clin. Lab. Anal..

[26] Wang Z., Cao Y.-C. (2014). An organic nanoparticle based high signal amplification immunoassay with improvement of nonspecific binding. J. Nanomed. Nanotechnol..

[27] Shao G., Wang J., Li Z., Saraf L., Wang W., Lin Y. (2011). Poly(dimethylsiloxane) microchip-based immunoassay with multiple reaction zones: Toward on-chip multiplex detection platform. Sens. Actuators B Chem..

[28] Xia H., Mathew B., John T., Hegab H., Feng J. (2013). Microfluidic based immunosensor for detection and purification of carbonylated proteins. Biomed. Microdevices.

[29] Brahim S., Narinesingh D., Guiseppi-Elie A. (2002). Interferent suppression using a novel polypyrrole-containing hydrogel in amperometric enzyme biosensors. Electroanalysis.

[30] Brahim S., Narinesingh D., Guiseppi-Elie A. (2001). Amperometric determination of cholesterol in serum using a biosensor of cholesterol oxidase contained within a polypyrrole-hydrogel membrane. Anal. Chim. Acta.

[31] Vashist S.K., Zheng D., Al-Rubeaan K., Luong J.H.T., Sheu F.-S. (2011). Advances in carbon nanotube based electrochemical sensors for bioanalytical applications. Biotechnol. Adv..

[32] Hudari F.F., Duarte E.H., Pereira A.C., Dall'Antonia L.H., Kubota L.T., Tarley C.R.T. (2013). Voltammetric method optimized by multi-response assays for the simultaneous measurements of uric acid and acetaminophen in urine in the presence of surfactant using MWCNT paste electrode. J. Electroanal. Chem..

[33] Kim J., Valdés-Ramírez G., Bandodkar A.J., Jia W., Martinez A.G., Ramírez J., Mercier P., Wang J. (2014). Non-invasive mouthguard biosensor for continuous salivary monitoring of metabolites. Analyst.

[34] Prabhakar A., Kumar Y.V.B.V., Tripathi S., Agrawal A. (2014). A novel, compact and efficient microchannel arrangement with multiple hydrodynamic effects for blood plasma separation. Microfluid. Nanofluidics.

[35] Kim D., Yun J.Y., Park S.-J., Lee S.S. (2009). Effect of microstructure on blood cell clogging in blood separators based on capillary action. Microsyst. Technol..

[36] Grieshaber D., MacKenzie R., Vörös J., Reimhult E. (2008). Electrochemical biosensors—Sensor principles and architectures. Sensors.

[37] Zhang X., Ju H., Wang J. (2008). Electrochemical Sensors, Biosensors and Their Biomedical Applications.

[38] Choi S., Goryll M., Sin L.Y.M., Wong P.K., Chae J. (2011). Microfluidic-based biosensors toward point-of-care detection of nucleic acids and proteins. Microfluid. Nanofluidics.

[39] Botasini S., Heijo G., Méndez E. (2013). Toward decentralized analysis of mercury (II) in real samples. A critical review on nanotechnology-based methodologies. Anal. Chim. Acta.

[40] Siegrist J., Gorkin R., Bastien M., Stewart G., Peytavi R., Kido H., Bergeron M., Madou M. (2010). Validation of a centrifugal microfluidic sample lysis and homogenization platform for nucleic acid extraction with clinical samples. Lab Chip.

[41] Spiegel H. (1984). Clinical Biochemistry V3: Contemporary Theories and Techniques.

[42] Herold K.E. (2009). Avraham Rasooly. Lab on a Chip Technology: Fabrication and Microfluidics.

[43] Newman J.D., Turner A.P.F. (2005). Home blood glucose biosensors: A commercial perspective. Biosens. Bioelectron..

[44] Matsumoto T., Furusawa M., Fujiwara H., Matsumoto Y., Ito N. (1998). A micro-planar amperometric glucose sensor unsusceptible to interference species. Sens. Actuators B Chem..

[45] Matsumoto T., Ohashi A., Ito N., Fujiwara H., Matsumoto T. (2001). A long-term lifetime amperometric glucose sensor with a perfluorocarbon polymer coating. Biosens. Bioelectron..

[46] Li Z., Liu M., Fan L., Ke H., Luo C., Zhao G. (2014). A highly sensitive and wide-ranged electrochemical zinc(II) aptasensor fabricated on core-shell SiO_2_-Pt@meso-SiO_2_. Biosens. Bioelectron..

[47] Makhaeva G.F., Malygin V.V., Strakhova N.N., Sigolaeva L.V., Sokolovskaya L.G., Eremenko A.V., Kurochkin I.N., Richardson R.J. (2007). Biosensor assay of neuropathy target esterase in whole blood as a new approach to OPIDN risk assessment: Review of progress. Hum. Exp. Toxicol..

[48] Noroozifar M., Khorasani-Motlagh M., Parizi M.B., Akbari R. (2013). Highly sensitive electrochemical detection of dopamine and uric acid on a novel carbon nanotube-modified ionic liquid-nanozeolite paste electrode. Ionics.

[49] Chen G., Wang Y., Yang P. (2005). Amperometric biosensor coupled to capillary electrophoresis for glucose determination. Microchim. Acta.

[50] Park B.-W., Zheng R., Ko K.-A., Cameron B.D., Yoon D.-Y., Kim D.-S. (2012). A novel glucose biosensor using bi-enzyme incorporated with peptide nanotubes. Biosens. Bioelectron..

[51] Eguílaz M., Moreno-Guzmán M., Campuzano S., González-Cortés A., Yáñez-Sedeño P., Pingarrón J.M. (2010). An electrochemical immunosensor for testosterone using functionalized magnetic beads and screen-printed carbon electrodes. Biosens. Bioelectron..

[52] Xue C., Han Q., Wang Y., Wu J., Wen T., Wang R., Hong J., Zhou X., Jiang H. (2013). Amperometric detection of dopamine in human serum by electrochemical sensor based on gold nanoparticles doped molecularly imprinted polymers. Biosens. Bioelectron..

[53] Yang H., Li Z., Wei X., Huang R., Qi H., Gao Q., Li C., Zhang C. (2013). Detection and discrimination of alpha-fetoprotein with a label-free electrochemical impedance spectroscopy biosensor array based on lectin functionalized carbon nanotubes. Talanta.

[54] Erden P.E., Zeybek B., Pekyardimc Ş., Kiliç E. (2013). Amperometric carbon paste enzyme electrodes with Fe_3_O_4_ nanoparticles and 1,4-Benzoquinone for glucose determination. Artif. Cells Nanomed. Biotechnol..

[55] Xu M., Luo X., Davis J.J. (2013). The label free picomolar detection of insulin in blood serum. Biosens. Bioelectron..

[56] Bertok T., Klukova L., Sediva A., Kasák P., Semak V., Micusik M., Omastova M., Chovanová L., Vlček M., Imrich R. (2013). Ultrasensitive impedimetriclectin biosensors with efficient antifouling properties applied in glycoprofiling of human serum samples. Anal. Chem..

[57] Pilehvar S., Ahmad Rather J., Dardenne F., Robbens J., Blust R., de Wael K. (2014). Carbon nanotubes based electrochemical aptasensing platform for the detection of hydroxylated polychlorinated biphenyl in human blood serum. Biosens. Bioelectron..

[58] Mathur A., Blais S., Goparaju C.M.V., Neubert T., Pass H., Levon K. (2013). Development of a Biosensor for Detection of Pleural Mesothelioma Cancer Biomarker Using Surface Imprinting. PLoS One.

[59] Cheemalapati S., Devadas B., Chen S.-M. (2014). Highly sensitive and selective determination of pyrazinamide at poly-l-methionine/reduced graphene oxide modified electrode by differential pulse voltammetry in human blood plasma and urine samples. J. Colloid Interface Sci..

[60] Bhansali S., Chornokur G., Arya S.K., Phelan C., Tanner R. (2011). Impedance-based miniaturized biosensor for ultrasensitive and fast prostate-specific antigen detection. J. Sens..

[61] Madasamy T., Pandiaraj M., Balamurugan M., Bhargava K., Sethy N.K., Karunakaran C. (2014). Copper, zinc superoxide dismutase and nitrate reductase coimmobilizedbienzymatic biosensor for the simultaneous determination of nitrite and nitrate. Biosens. Bioelectron..

[62] Rosales-Rivera L.C., Acero-Sánchez J.L., Lozano-Sánchez P., Katakis I., O'Sullivan C.K. (2012). Amperometric immunosensor for the determination of IgA deficiency in human serum samples. Biosens. Bioelectron..

[63] Luhana C., Bo X.-J., Ju J., Guo L.-P. (2012). A novel enzymatic glucose sensor based on Pt nanoparticles-decorated hollow carbon spheres-modified glassy carbon electrode. J. Nanoparticle Res..

[64] Hong C.-Y., Chen X., Liu T., Li J., Yang H.-H., Chen J.-H., Chen G.-N. (2013). Ultrasensitive electrochemical detection of cancer-associated circulating microRNA in serum samples based on DNA concatamers. Biosens. Bioelectron..

[65] Conneely G., Aherne M., Lu H., Guilbault G.G. (2007). Development of an immunosensor for the detection of testosterone in bovine urine. Anal. Chim. Acta.

[66] Radhapyari K., Kotoky P., Das M.R., Khan R. (2013). Graphene-polyanilinenanocomposite based biosensor for detection of antimalarial drug artesunate in pharmaceutical formulation and biological fluids. Talanta.

[67] Kannan P., Maiyalagan T., Sahoo N.G., Opallo M. (2013). Nitrogen doped graphenenanosheet supported platinum nanoparticles as high performance electrochemical homocysteine biosensors. J. Mater. Chem. B.

[68] Gan N., Wang L.-Y., Xu W.-M., Li T.-H., Jiang Q.-L. (2007). Electrochemical Immuno-Biosensor for the Rapid Determination of Nuclear Matrix Protein 22 (NMP22) antigen in Urine Samples by Co(III) Phthlocyanine/Fe_3_O_4_/Au Collide Coimmobilized Electrode. Chin. J. Anal. Chem..

[69] Yu D., Zeng Y., Qi Y., Zhou T., Shi G. (2012). A novel electrochemical sensor for determination of dopamine based on AuNPs@SiO_2_ core-shell imprinted composite. Biosens. Bioelectron..

[70] Xue Y., Sheng Z., Zhao H., Wu Z., Li X., He Y., Yuan Z. (2012). Electrochemical synthesis and characterization of a novel thiazole-based copolymer and its application in biosensor. Electrochim. Acta.

[71] Liu X., Xie L., Li H. (2012). Electrochemical biosensor based on reduced graphene oxide and Au nanoparticles entrapped in chitosan/silica sol-gel hybrid membranes for determination of dopamine and uric acid. J. Electroanal. Chem..

[72] Ballesta-Claver J., Rodríguez-Gómez R., Capitán-Vallvey L.F. (2013). Disposable biosensor based on cathodic electrochemiluminescence of tris(2,2-bipyridine)ruthenium(II) for uric acid determination. Anal. Chim. Acta.

[73] BallestaClaver J., Valencia Mirón M.C., Capitán-Vallvey L.F. (2009). Disposable electrochemiluminescent biosensor for lactate determination in saliva. Analyst.

[74] Bunyakul N., Promptmas C., Baeumner A.J. (2014). Microfluidic biosensor for cholera toxin detection in fecal samples. Anal. Bioanal. Chem..

[75] Yang X., Forouzan O., Brown T.P., Shevkoplyas S.S. (2012). Integrated separation of blood plasma from whole blood for microfluidic paper-based analytical devices. Lab Chip.

[76] Lin H.K., Zheng S., Williams A.J., Balic M., Groshen S., Scher H.I., Fleisher M., Stadler W., Datar R.H., Tai Y.-C. (2010). Portable filter-based microdevice for detection and characterization of circulating tumor cells. Clin. Cancer Res..

[77] Castro-López V., Elizalde J., Pacek M., Hijona E., Bujanda L. (2014). A simple and portable device for the quantification of TNF-α in human plasma by means of on-chip magnetic bead-based proximity ligation assay. Biosens. Bioelectron..

[78] Lee B.S., Lee J.-N., Park J.-M., Lee J.-G., Kim S., Cho Y.-K., Ko C. (2009). A fully automated immunoassay from whole blood on a disc. Lab Chip.

[79] Lee M.G., Shin J.H., Bae C.Y., Choi S., Park J.-K. (2013). Label-free cancer cell separation from human whole blood using inertial microfluidics at low shear stress. Anal. Chem..

[80] Kuntaegowdanahalli S.S., Bhagat A.A.S., Kumar G., Papautsky I. (2009). Inertial microfluidics for continuous particle separation in spiral microchannels. Lab Chip.

[81] Yan S., Zhang J., Alici G., Du H., Zhu Y., Li W. (2014). Isolating plasma from blood using a dielectrophoresis-active hydrophoretic device. Lab Chip.

[82] Kersaudy-Kerhoas M., Dhariwal R., Desmulliez M.P.Y., Jouvet L. (2010). Hydrodynamic blood plasma separation in microfluidic channels. Microfluid. Nanofluidics.

[83] Takagi J., Yamada M., Yasuda M., Seki M. (2005). Continuous particle separation in a microchannel having asymmetrically arranged multiple branches. Lab Chip.

[84] Petersson F., Nilsson A., Holm C., Jönsson H., Laurell T. (2004). Separation of lipids from blood utilizing ultrasonic standing waves in microfluidic channels. Analyst.

[85] Helton K.L., Nelson K.E., Fu E., Yager P. (2008). Conditioning saliva for use in a microfluidic biosensor. Lab Chip.

[86] Mohamed H., McCurdy L.D., Szarowski D.H., Duva S., Turner J.N., Caggana M. (2004). Development of a rare cell fractionation device: Application for cancer detection. IEEE Trans. Nanobiosci..

[87] Crowley T.A., Pizziconi V. (2005). Isolation of plasma from whole blood using planar microfilters for lab-on-a-chip applications. Lab Chip.

[88] Van Delinder V., Groisman A. (2006). Separation of plasma from whole human blood in a continuous cross-flow in a molded microfluidic device. Anal. Chem..

[89] Mulvaney S.P., Cole C.L., Kniller M.D., Malito M., Tamanaha C.R., Rife J.C., Stanton M.W., Whitman L.J. (2007). Rapid, femtomolar bioassays in complex matrices combining microfluidics and magnetoelectronics. Biosens. Bioelectron..

[90] Dimov I.K., Garcia-Cordero J.L., O'Grady J., Poulsen C.R., Viguier C., Kent L., Daly P., Lincoln B., Maher M., O'Kennedy R. (2008). Integrated microfluidic tmRNA purification and real-time NASBA device for molecular diagnostics. Lab Chip.

[91] Reboud J., Bourquin Y., Wilson R., Pall G.S., Jiwaji M., Pitt A.R., Graham A., Waters A.P., Cooper J.M. (2012). Shaping acoustic fields as a toolset for microfluidic manipulations in diagnostic technologies. Proc. Natl. Acad. Sci. USA.

[92] Oblath E.A., Henley W.H., Alarie J.P., Ramsey J.M. (2013). A microfluidic chip integrating DNA extraction and real-time PCR for the detection of bacteria in saliva. Lab Chip.

[93] Marshall L.A., Wu L.L., Babikian S., Bachman M., Santiago J.G. (2012). Integrated printed circuit board device for cell lysis and nucleic acid extraction. Anal. Chem..

[94] Wang C.-H., Lien K.-Y., Hung L.-Y., Lei H.-Y., Lee G.-B. (2012). Integrated microfluidic system for the identification and multiple subtyping of influenza viruses by using a molecular diagnostic approach. Microfluid. Nanofluidics.

[95] Omiatek D.M., Santillo M.F., Heien M.L., Ewing A.G. (2009). Hybrid capillary-microfluidic device for the separation, lysis, and electrochemical detection of vesicles. Anal. Chem..

[96] Lam B., Fang Z., Sargent E.H., Kelley S.O. (2012). Polymerase chain reaction-free, sample-to-answer bacterial detection in 30 minutes with integrated cell lysis. Anal. Chem..

[97] Yang S., Ündar A., Zahn J.D. (2006). A microfluidic device for continuous, real time blood plasma separation. Lab Chip.

[98] Fan R., Vermesh O., Srivastava A., Yen B.K.H., Qin L., Ahmad H., Kwong G.A., Liu C.-C., Gould J., Hood L. (2008). Integrated barcode chips for rapid, multiplexed analysis of proteins in microliter quantities of blood. Nat. Biotechnol..

[99] Park J.-S., Jung H.-I. (2009). Multiorifice flow fractionation: Continuous size-based separation of microspheres using a series of contraction/expansion microchannels. Anal. Chem..

[100] Zhang J., Yan S., Sluyter R., Li W., Alici G., Nguyen N.-T. (2014). Inertial particle separation by differential equilibrium positions in a symmetrical serpentine micro-channel. Sci. Rep..

[101] Wang X., Zhou J., Papautsky I. (2013). Vortex-aided inertial microfluidic device for continuous particle separation with high size-selectivity, efficiency, and purity. Biomicrofluidics.

[102] Parichehreh V., Medepallai K., Babbarwal K., Sethu P. (2013). Microfluidic inertia enhanced phase partitioning for enriching nucleated cell populations in blood. Lab Chip.

[103] MacH A.J., Kim J.H., Arshi A., Hur S.C., di Carlo D. (2011). Automated cellular sample preparation using a Centrifuge-on-a-Chip. Lab Chip.

[104] Zhang J.Y., Do J., Premasiri W.R., Ziegler L.D., Klapperich C.M. (2010). Rapid point-of-care concentration of bacteria in a disposable microfluidic device using meniscus dragging effect. Lab Chip.

[105] Yamada M., Seki M. (2005). Hydrodynamic filtration for on-chip particle concentration and classification utilizing microfluidics. Lab Chip.

[106] Yamada M., Kano K., Tsuda Y., Kobayashi J., Yamato M., Seki M., Okano T. (2007). Microfluidic devices for size-dependent separation of liver cells. Biomed. Microdevices.

[107] Morijiri T., Sunahiro S., Senaha M., Yamada M., Seki M. (2011). Sedimentation pinched-flow fractionation for size- and density-based particle sorting in microchannels. Microfluid. Nanofluidics.

[108] Yamada M., Nakashima M., Seki M. (2004). Pinched flow fractionation: Continuous size separation of particles utilizing a laminar flow profile in a pinched microchannel. Anal. Chem..

[109] Weigl B.H., Bardell R.L., Kesler N., Morris C.J. (2001). Lab-on-a-chip sample preparation using laminar fluid diffusion interfaces computational fluid dynamics model results and fluidic verification experiments. Anal. Bioanal. Chem..

[110] Gao J., Sin M.L.Y., Liu T., Gau V., Liao J.C., Wong P.K. (2011). Hybrid electrokinetic manipulation in high-conductivity media. Lab Chip.

[111] Krishnan R., Sullivan B.D., Mifflin R.L., Esener S.C., Heller M.J. (2008). Alternating current electrokinetic separation and detection of DNA nanoparticles in high-conductance solutions. Electrophoresis.

[112] Park S., Zhang Y., Wang T.-H., Yang S. (2011). Continuous dielectrophoretic bacterial separation and concentration from physiological media of high conductivity. Lab Chip.

[113] Gao J., Riahi R., Sin M.L.Y., Zhang S., Wong P.K. (2012). Electrokinetic focusing and separation of mammalian cells in conductive biological fluids. Analyst.

[114] Nilsson A., Petersson F., Jönsson H., Laurell T. (2004). Acoustic control of suspended particles in micro fluidic chips. Lab Chip.

[115] Chung T.D., Kim H.C. (2007). Recent advances in miniaturized microfluidic flow cytometry for clinical use. Electrophoresis.

[116] Vitorino R., Guedes S., Manadas B., Ferreira R., Amado F. (2012). Toward a standardized saliva proteome analysis methodology. J. Proteomics.

[117] Gillers S., Atkinson C.D., Bartoo A.C., Mahalanabis M., Boylan M.O., Schwartz J.H., Klapperich C., Singh S.K. (2009). Microscale sample preparation for PCR of *C. difficile* infected stool. J. Microbiol. Methods.

[118] Silva B.V.M., Cavalcanti I.T., Mattos A.B., Moura P., Sotomayor M.D.P.T., Dutra R.F. (2010). Disposable immunosensor for human cardiac troponin T based on streptavidin-microsphere modified screen-printed electrode. Biosens. Bioelectron..

[119] Zhou F., Lu M., Wang W., Bian Z.-P., Zhang J.-R., Zhu J.-J. (2010). Electrochemical immunosensor for simultaneous detection of dual cardiac markers based on a poly(dimethylsiloxane)-gold nanoparticles composite microfluidic chip: A proof of principle. Clin. Chem..

[120] Dimov I.K., Basabe-Desmonts L., Garcia-Cordero J.L., Ross B.M., Ricco A.J., Lee L.P. (2011). Stand-alone self-powered integrated microfluidic blood analysis system (SIMBAS). Lab Chip.

[121] Drummer O.F. (2006). Drug Testing in Oral Fluid. Clin. Biochem. Rev..

